# P300 Analysis Using High-Density EEG to Decipher Neural Response to rTMS in Patients With Schizophrenia and Auditory Verbal Hallucinations

**DOI:** 10.3389/fnins.2020.575538

**Published:** 2020-11-20

**Authors:** Romain Aubonnet, Ovidiu C. Banea, Roberta Sirica, Eric M. Wassermann, Sahar Yassine, Deborah Jacob, Brynja Björk Magnúsdóttir, Magnús Haraldsson, Sigurjon B. Stefansson, Viktor D. Jónasson, Eysteinn Ívarsson, Aron D. Jónasson, Mahmoud Hassan, Paolo Gargiulo

**Affiliations:** ^1^Institute of Biomedical and Neural Engineering/Medical Technology Center, Reykjavik University, Reykjavik, Iceland; ^2^Clinical Neurophysiology Unit, Neurology Department, National University Hospital of Iceland, Reykjavik, Iceland; ^3^National Institute of Neurological Disorders and Stroke, Bethesda, MD, United States; ^4^NeuroKyma, Rennes, France; ^5^Department of Psychiatry, National University Hospital, Reykjavik, Iceland; ^6^Department of Psychology, Reykjavik University, Reykjavik, Iceland; ^7^University of Rennes 1, LTSI, Rennes, France; ^8^Department of Science, National University Hospital, Reykjavik, Iceland

**Keywords:** high-density EEG, TMS (repetitive transcranial magnetic stimulation), P300, schizophrenia, spectral analysis, temporal analysis, brain connectivity

## Abstract

Schizophrenia is a complex disorder about which much is still unknown. Potential treatments, such as transcranial magnetic stimulation (TMS), have not been exploited, in part because of the variability in behavioral response. This can be overcome with the use of response biomarkers. It has been however shown that repetitive transcranial magnetic stimulation (rTMS) can the relieve positive and negative symptoms of schizophrenia, particularly auditory verbal hallucinations (AVH). This exploratory work aims to establish a quantitative methodological tool, based on high-density electroencephalogram (HD-EEG) data analysis, to assess the effect of rTMS on patients with schizophrenia and AVH. Ten schizophrenia patients with drug-resistant AVH were divided into two groups: the treatment group (TG) received 1 Hz rTMS treatment during 10 daily sessions (900 pulses/session) over the left T3-P3 International 10-20 location. The control group (CG) received rTMS treatment over the Cz (vertex) EEG location. We used the P300 oddball auditory paradigm, known for its reduced amplitude in schizophrenia with AVH, and recorded high-density electroencephalography (HD-EEG, 256 channels), twice for each patient: pre-rTMS and 1 week post-rTMS treatment. The use of HD-EEG enabled the analysis of the data in the time domain, but also in the frequency and source-space connectivity domains. The HD-EEG data were linked with the clinical outcome derived from the auditory hallucinations subscale (AHS) of the Psychotic Symptom Rating Scale (PSYRATS), the Quality of Life Scale (QoLS), and the Depression, Anxiety and Stress Scale (DASS). The general results show a variability between subjects, independent of the group they belong to. The time domain showed a higher N1-P3 amplitude post-rTMS, the frequency domain a higher power spectral density (PSD) in the alpha and beta bands, and the connectivity analysis revealed a higher brain network integration (quantified using the participation coefficient) in the beta band. Despite the small number of subjects and the high variability of the results, this work shows a robust data analysis and an interplay between morphology, spectral, and connectivity data. The identification of a trend post-rTMS for each domain in our results is a first step toward the definition of quantitative neurophysiological parameters to assess rTMS treatment.

## 1. Introduction

Hallucinations are sensory perceptions occurring in the absence of an external stimulus. Auditory verbal hallucinations (AVH) are positive psychotic symptoms of schizophrenia and a diagnostic feature in the pathology, occurring in an estimated 60–70% of people with this disorder. An increased interaction among the auditory-language and striatal brain regions occurs while patients hallucinate (Ćurčić Blake et al., 2017). Patients with AVH present evidence of structural brain alterations associated with these perceptions, such as reduced gray matter volume in the superior temporal gyrus (Kasai et al., 2003), including the primary auditory cortex, and abnormal connectivity among the temporal, prefrontal, and anterior cingulate regions (Homan, 2013; Ćurčić Blake et al., 2017). Among the empirically supported theories of the origin of AVH, are a misinterpretation of inner speech (Frith and Done, 1988) and aberrant activation of the auditory cortex (Dierks et al., 1999). Almost one-third of patients with positive psychotic schizophrenia present treatment resistant symptoms (Howes et al., 2009) and there is a compelling need for novel treatments.

Transcranial magnetic stimulation (TMS) is a non-invasive method used over the past 25 years in the treatment of neurobehavioral disorders (Stanford et al., 2008). It uses an alternating magnetic field to induce an electrical current in the brain, depolarizing neurons and generating action potentials. Wassermann et al. (1996) and Chen et al. (1997) reported that 1 Hz repetitive TMS (rTMS) reduces the excitability of cortical neurons in healthy individuals. Based on these effects, Hoffman et al. (1999) hypothesized that 1 Hz rTMS delivered to the left temporoparietal cortex reduced activity in receptive language areas associated with AVH in patients with schizophrenia. Neuroimaging studies of AVH showed an increased activation in the absence of an external stimulus in the left primary auditory cortex of subjects with this symptom (Kompus et al., 2011).

Our goal was to establish a methodological tool to quantitatively assess the cognitive processes of people suffering with AVH. We aimed to develop an hypothesis that can validate psychometric results with event related potential (ERP) morphology (time domain), power spectral density (frequency domain), and brain connectivity in patients undergoing 10 sessions of low-frequency rTMS.

To date, the mechanism of the effect of rTMS on AVH has only been inferred from the hypothesis of left temporoparietal cortex dysfunction and the behavioral response is variable from patient to patient. Dozens of studies have used inhibitory low frequency rTMS over the T3-P3 EEG location as a treatment for pharmaco-resistant AVH with the effects measured mainly with psychometric scales (Lefaucheur et al., 2014; Slotema et al., 2014). Physiological measures linked to specific brain areas and biomarkers of target engagement and response are needed to optimize treatment. Indeed, response biomarkers are essential as predictors of treatment where behavioral outcomes can be variable. They may also be very useful for rTMS treatments, where multiple parameters, including frequency, train length, intensity, duration, and treatment schedule can all influence effectiveness and should be optimized before full-scale clinical trials are attempted. Past studies indicate a relation between different frequency bands and cognitive processes (Klimesch et al., 1998). The power spectral density (PSD) changes observed in response to attentional demands can be of interest to monitor patients with schizophrenia behavior. Electroencephalographic (EEG) measures, including spectral density and evoked potentials (Barr et al., 2011), have been used as measures of the physiological response to TMS treatment. For instance, it has been observed that rTMS to the dorsolateral prefrontal area increased the P300 response in patients with schizophrenia, but not healthy controls (Lin et al., 2018).

The P300 first described by Sutton et al. (1965), mostly studied as a parameter of voluntary attention (Mazaheri and Picton, 2005), is the leading Event Related Potential (ERP) correlate of target discrimination (Mazaheri and Picton, 2005) and it has been largely employed to characterize schizophrenia (Jeon and Polich, 2003). Previous studies have found that patients with auditory hallucinations exhibit reduced P300 amplitudes (Jeon and Polich, 2003; Bramon et al., 2004; Fisher et al., 2014). Many works based on P300 also analyzed N100, the negative deflection that occurs approximately 100 ms after the auditory stimulus, noting a relation with working memory (Lijffijt et al., 2009). The mean amplitudes of the auditory N100 and P300 responses are decreased in patients with schizophrenia in comparison to healthy participants (Ogura et al., 1991; Ford et al., 2001; Earls et al., 2016).

Here, we compared alterations in the P300 response after left temporal and vertex [used as a control in previous studies with schizophrenia and AVH (Nyffeler et al., 2006; Nowak et al., 2008; Loo et al., 2010)] TMS in patients with schizophrenia using three different approaches : time, frequency, and source-space connectivity. Patients also underwent a battery of neurobehavioral and tests before and after treatment.

We remained descriptive in our analysis before and after treatment, at group and single levels.

## 2. Materials and Methods

### 2.1. Participants

**Figure 1:**
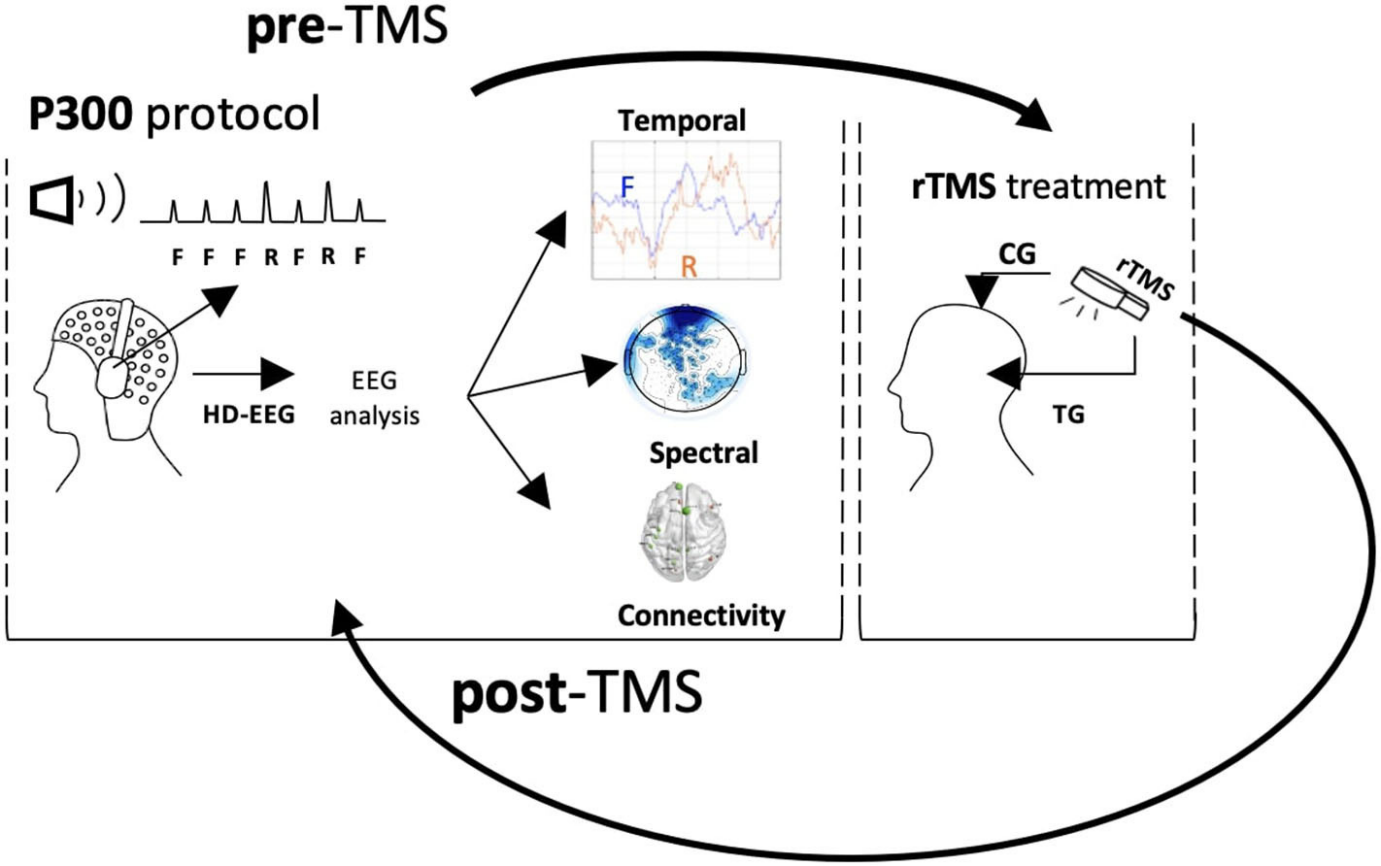
Data acquisition and processing workflow.

**Figure 2:**
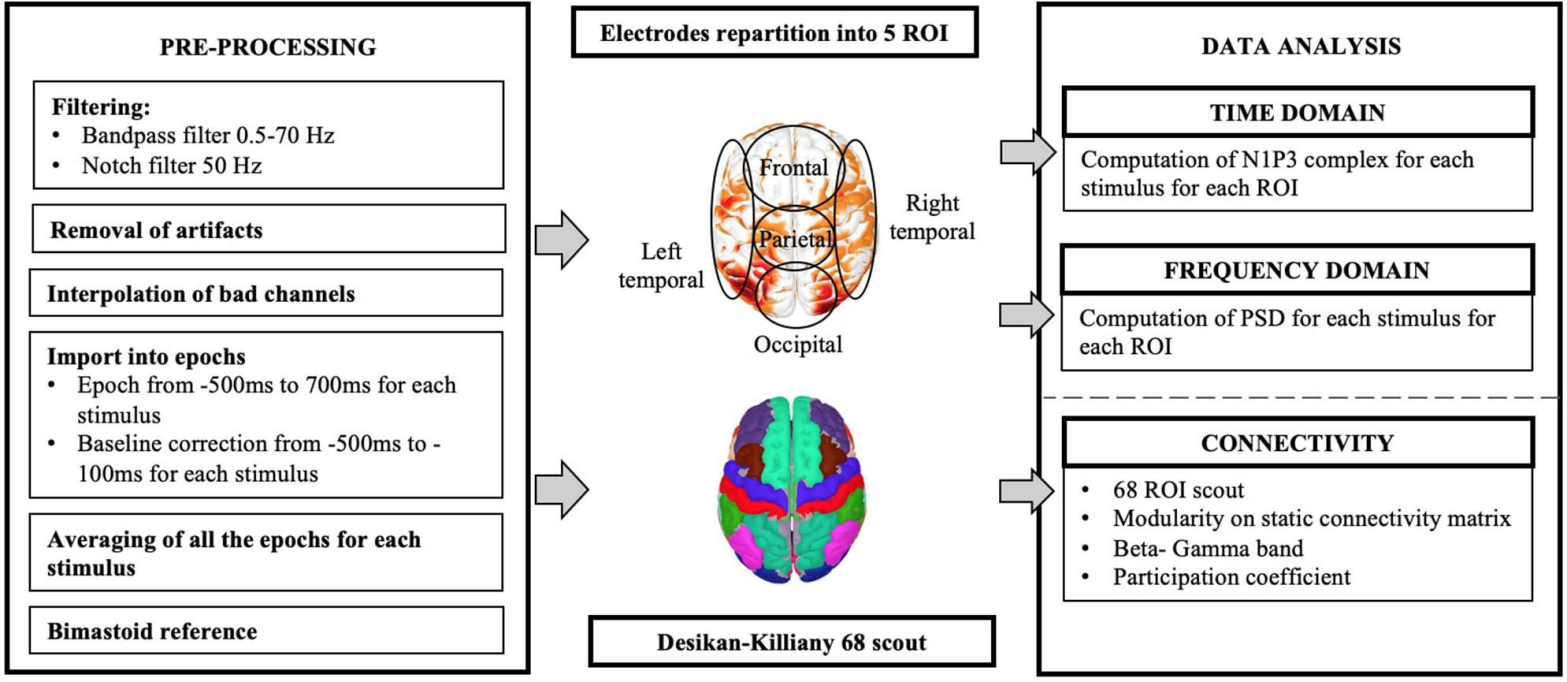
Pre-processing and analysis workflow.

The patients were recruited from the psychiatric wards and outpatient clinics of the National Hospital of Iceland. They were diagnosed with schizophrenia, following the ICD-10 (International Classification of Diseases, Tenth Revision, Clinical Modification) schizophrenia classification (F20). Only those still experiencing persistent AVH after finishing at least two 6–8 week drug treatments were selected. Patients were excluded if they had history of seizures, were using cannabis or drinking more than three units of alcohol daily, were using any other illegal drugs within 1 month prior to the beginning of the study, or showing TMS contraindications during the pre-treatment interview (Rossi et al., 2011). Permission from the Health Research Ethics Committee at the University Hospital of Iceland was obtained (approval no. 21.2018). Ten patients (7 men and 3 women, mean age = 32, SD = 6.41) were selected for the study. All of them were taking medications. Table 1 sums up the patient information. The patients were randomly assigned into two groups. Five patients (four men and one woman, mean age 35.2, SD = 5.12,range 30–48) were included in the active treatment group (TG). They received ten daily sessions of 15 min 1 Hz frequency rTMS (900 pulses/session) at 100% of abductor pollicis brevis resting motor threshold (RMT) applied at T3-P3 location. Five patients (three men and two women, mean age 29.6, SD = 3.92, range 26–39) were included in the control group (CG) and received rTMS at 100% RMT to the vertex of CG 10-20 location. The EEG and psychometric data were acquired twice in each patient group; before the rTMS treatment (pre-treatment) and within 1 week after completing ten sessions of rTMS treatment (post-treatment). This produced 20 datasets: five pre-TMS TG, five post-TMS TG, five pre-TMS CG, and five post-TMS CG. Figure 1 shows the experimental set-up and workflow designed for this study. Figure 2 shows the pre-processing and data analysis pipeline used for this study.

**Table 1:** Socio-demographic information.

**Group**	**Id**	**Gender**	**Medication**	**Diagnosis**
TG	T1	M	Clozapine, Fluoxetine, Bupropion	Paranoid Schizophrenia
TG	T2	M	Clozapine, Olanzapine, Perphenazine, Alprazolam, Levomepromazine, Oxazepam and Melatonin	Paranoid Schizophrenia
TG	T3	F	Sertraline, Quetiapine, Pregabalin and Zopiclone	Schizoaffective Disorder Depressive type
TG	T4	M	Clozapine and Flupenthixol	Paranoid Schizophrenia
TG	T5	M	Clozapine, Amisulpiride, Propranolol and Clonazepam	Paranoid Schizophrenia
CG	C1	M	Paliperidone, Quetiapine and Perphenazine	Paranoid Schizophrenia
CG	C2	F	Clozapine, Flupenthixol, Zopiclone, Mirtazapine, Escitalopram, Metoprolol and Chlorpromazine	Paranoid Schizophrenia
CG	C3	F	Aripiprazole, Olanzapine, Chlorprothixene and Pregabalin	Paranoid Schizophrenia
CG	C4	M	Clozapine, Olanzapine, Bupropion and Propranolol	Paranoid Schizophrenia
CG	C5	M	Clozapine, Pregabalin, Amisulpride	Hebephrenic Schizophrenia

*(Group : TG, T3-P3 group; CG, Cz group. Gender : M, man; F, woman.)*.

### 2.2. Psychometric Data

Three scales were used to collect clinical information pre- and post-treatment.

#### 2.2.1. PSYRATS

Psychotic Symptom Rating Scales (PSYRATS) auditory hallucinations subscale (AHS) is an interview measuring auditory hallucinations using 11 items rated on a five-point ordinal scale (0–4). The scale measures the severity of AVH for the past week in 11 dimensions which are: frequency, duration, location, loudness, beliefs about origin, negative content, intensity of negative content, amount of distress, intensity of distress, disruption of life, and control. PSYRATS has shown excellent inter-rater reliability and good discriminant and convergent validity for both chronic and first episode psychosis (Haddock et al., 1999; Drake et al., 2007).

#### 2.2.2. Quality of Life Scale (QoLS)

Quality of life was assessed with a 16 item self-report scale, consisting of five conceptual domains of quality of life: material and physical well-being, relationships with other people, social community and civic activities, personal development and fulfillment, and recreation. The scale has been shown to have good test-retest reliability and good convergent and discriminant validity (Flanagan, 1978).

#### 2.2.3. Depression Anxiety Stress Scales (DASS)

The DASS is a measure of mental health focusing on the three traits of depression, anxiety, and stress. It consists of 42 items, rated on a four point Likert type scale of how much that symptom occurred in the last week. In clinical samples the scale has shown excellent internal consistency and temporal stability as well as excellent discriminant validity and good convergent validity (Brown et al., 1997).

### 2.3. P300 Recordings

P300 was measured with an auditory oddball paradigm attention task. The recordings took place between 11h00 and 14h00 for a duration of 1 h. The subjects sat with their eyes closed. The frequent (F) and the rare (R) auditory stimuli were presented binaurally through headphones at an interstimulus interval between tones of constant 1.1 s. The loudness was adjusted for each participant. For each subject, there was one trial of 200 tones, comporting a random tone occurrence with a probability of 0.2, leading to 160 frequent tones and 40 rare (Marcu et al., 2020). We required the participants to focus on the rare stimuli without counting or moving a finger.

### 2.4. EEG Pre-processing and Analysis

The EEG was recorded using a 256 channel system (ANT Neuro, Netherlands) with an electrooculogram (EOG) electrode placed below the right eye and a ground electrode placed on the left side of the neck. Data pre-processing and analysis were performed with Brainstorm (Tadel et al., 2011) and MATLAB 2018b (MathWorks, Inc., Natick, 158 Massachusetts, USA).

#### 2.4.1. Pre-processing

The data were sampled at 1,024 Hz and re-referenced to the average of left and right mastoid electrodes (R19R, L19L). A bandpass filter was set between 0.5 and 70 Hz and notch filter from 49 to 51 Hz was used to remove undesired monomorphic artifacts from 50 Hz mains electricity. Bad channels were manually removed when EEG voltage was higher than ±80 μV; if more than 10% of the channels showed too much noise or incorrect signal, the whole trial was rejected. The signals were digitized in epochs of 1,200 ms, starting 500 ms before the presentation of each auditory stimulus (–500 to +700 ms). Baseline correction was performed using pre-stimulus 500 ms to pre-stimulus 100 ms window and channels marked as bad were removed and interpolated. Individual trials were visually inspected and rejected when indicative of excessive muscle activity, eye movements, or other artifacts.

#### 2.4.2. Data Analysis

##### 2.4.2.1. Time Domain

N100-P300 complex values of both frequent and rare stimuli were calculated and plotted via MATLAB 2018b for each subject (pre- and post-treatment for patients groups).

The scalp was divided into 5 regions of interest (ROI), see Figure 2. The 254 electrodes were partitioned as follows: 80 channels for the Frontal region (F), 59 for the Parietal region (P), 69 for the Occipital region (O), 23 for Right Temporal lobe (RT), and 23 for Left Temporal lobe (LT) (Schartner et al., 2015).

N1-P3 wave signals were calculated for the entire N100-P300 complex from the average of channels of every ROI as the difference between the most negative voltage value within time range of 80–150 ms (N100) and the most positive voltage value within time range of 250–500 ms (P300).

The differences between frequent and rare stimulus and pre- and post-treatment were also computed and plotted.

##### 2.4.2.2. Frequency Domain

The power spectral density (PSD) was computed for each epoch with Welch's method, using Brainstorm, with the following frequency bands: delta (0.5–4 Hz), theta (4–8 Hz), alpha (8–13 Hz), beta (13–30 Hz), gamma (30–70 Hz). The PSD has been divided by the associated bandwidth for each frequency band.

Using the same scalp division as that of the time analysis, the PSD of electrodes within the same ROI were averaged for frequent and rare stimuli, pre- and post-treatment for each subject.

The PSD difference post-pre treatment and frequent—rare were computed for each subject.

##### 2.4.2.3. Connectivity

The connectivity has been computed at the cortical level using the "EEG source connectivity" method. It consists of estimating the brain sources (over 68 regions of interest—ROI—) and then computing the statistical coupling between these reconstructed sources. The weighted minimum norm estimate (wMNE) and the Phase Locking Value (PLV) were used to solve the inverse problem and compute the functional connectivity, respectively. This choice was based on previous comparative studies showing good performance of this combination on simulated and real data. (Hassan et al., 2014, 2017; Hassan and Wendling, 2018). The analysis has been performed only on the beta and gamma bands, due to window length constraints (here 700 ms). The source-space networks were estimated for each trial, subject and conditions. To compare between conditions, the networks were quantified using network measures that allow the extraction of the topological properties of the networks. We made the choice here to focus on network integration as it is the most consistent network feature that changes due to electric/magnetic stimulation (Modolo et al., 2020) or brain disorders (Stam, 2014). The network integration reflects the ability of the brain network to integrate information form different and distant brain regions, a key feature of efficient information processing. To quantify the network integration, we used the participation coefficient (PC), to calculate the interactions between brain modules (distant sub-networks), on the thresholded connectivity matrices (here 20%). We used the brain connectivity toolbox (BCT) (Rubinov and Sporns, 2010) to compute the PC (http://www.brain-connectivity-toolbox.net/).

## 3. Results

### 3.1. Group Results

The analysis for each individual revealed general consistent results. The results picturing the evolution (increase or decrease) of the neurophysiological and psychometric data and are detailed in Table 2 for the TG, and in Table 3 for the CG. The associated numerical values are detailed in Table 4 for the TG and in Table 5 for the CG. The analysis of the psychometric tests revealed that four out of five subjects in TG (Tables 2, 4) and three out of five subjects in CG (Tables 3, 5) felt improved condition after the treatment, whereas the other subjects remained neutral or reported worse psychometric scores. In the time domain analysis, the N1-P3 amplitude was globally higher post-treatment than pre-treatment, for six subjects, two in TG (Tables 2, 4) and four in CG (Tables 3, 5). The PSD increased post-treatment mainly for the alpha band and beta band globally, for six subjects as well, two in TG (Tables 2, 4) and four in CG (Tables 3, 5). No trends were detectable for the gamma and theta bands. In several subjects, the right temporal area showed an opposite behavior compared to the other regions. The connectivity results showed an increased network integration (increase in participation coefficient) during post-treatment for frequent, for the beta band especially, for seven subjects, four in CG (Tables 3, 5), three in TG (Tables 2, 4). Due to the small sample size and high variability of the results, we will discuss selected study cases individually. The following four patients were selected due to their interplay between psychometric score and neurophysiological results, independant of treatment. Two subjects (T2, in TG and C3, in CG) presented an improvement in the psychometric score post-TMS, and the two others presented a stagnation in the psychometric (C2, in CG) or a decrement (T5, in TG). The rest of the data are provided in the Supplementary Material. There were no significant changes on AVH severity measured with PSYRATS AHS, in QoL and DASS global scores after rTMS between TG and CG.

Table 2Treated Group: Increase (↑), decrease (↓), or constancy (–) of the value after treatment of N1-P3, Connectivity, Psychometric and Power spectral density (PSD) of the frequent (blue) and rare (orange) stimuli (F, frontal; P, parietal; O, occipital; LT, left temporal; RT, right temporal).

**Table:** 

	**N1-P3 amplitude (μV)**					**Connectivity**	**Psychometrics**		
	**F**	**P**	**O**	**LT**	**RT**	**Participation coefficient (%)**	**QoL**	**DASS**	**PSYRATS**
T1	↑↑	-↓	↑↓	↓↓	↑↓	↑	↑	-	↓
T2	↓↓	-↑	↓↓	↓↑	↓↓	↑	↑	↓	↓
T3	-↑	↑↑	↓↓	↓-	–	-	↑	↓	↓
T4	↑↑	↑↑	↑↑	↑↑	↑↑	-	-	-	↓
T5	↓↓	↓-	↓↓	↓↓	↓↓	↑	-	↑	↑

**Table:** 

	**Power spectral density**																			
	**PSD: THETA**					**PSD: ALPHA**					**PSD: BETA**					**PSD: GAMMA**				
	**F**	**P**	**O**	**LT**	**RT**	**F**	**P**	**O**	**LT**	**RT**	**F**	**P**	**O**	**LT**	**RT**	**F**	**P**	**O**	**LT**	**RT**
T1	-↓	↓↓	↓↓	↓↓	↓↓	↓↓	↓↓	↓↓	↓↓	↓↓	↓↓	↓-	↓↓	↓↓	↓↓	↑-	↑↑	-↓	↓↓	↑↑
T2	↓↓	↓↓	↓-	↓↓	↓↑	↑↑	-↑	↑↑	↑↑	↑↑	↓↓	↓↓	↓↓	↓↓	↓↓	↓↓	↓↓	↓↓	↓↓	↓↓
T3	↓↓	↓↓	↓↓	↓↓	↓↑	↓↓	↓↓	↓↓	↓↓	↓↓	↓↓	↓↓	↓↓	↓↓	↓↓	↑↑	↑↑	↑↑	↑↑	↑↑
T4	↓↓	↓↓	-↓	↑-	↓↓	↑↑	↑↑	↑↑	↑↑	↓↓	↑↑	↑↑	↑↑	↑↑	↓↓	↑↑	↑↑	↑↑	↑↑	↑↑
T5	↑↑	↑↑	↑↑	↑↑	↓-	↑↑	↑↑	↑↑	–	↓↓	↑↑	↑↑	↑↑	↑↑	↓↑	↑↑	↑↑	↑↑	↑↑	↓↓

*QoLS, Quality of Life Scale; DASS, Depression Anxiety Stress Scale; PSYRATS, Psychotic Symptom Rating Scales*.

Table 3Control Group: Increase (↑), decrease (↓) or constancy (-) of the value after treatment of N1-P3, Connectivity, Psychometric and Power Spectral Density (PSD) of the frequent (blue) and rare (orange) stimuli(F, frontal; P, parietal; O, occipital; LT, left temporal; RT, right temporal).

**Table:** 

	**N1-P3 amplitude (μV)**					**Connectivity**	**Psychometrics**		
	**F**	**P**	**O**	**LT**	**RT**	**Participation coefficient (%)**	**QoL**	**DASS**	**PSYRATS**
C1	↓↑	↓↑	↓ ↑	↓↑	↓↑	-	-	↓	-
C2	–	↓↓	-↑	↑↑	↑↑	↑	-	-	-
C3	↑↑	↑↑	↑↑	↑↓	↑↓	↑	↑	↓	-
C4	↓↓	↓↓	↓↓	↓↓	↓↓	↑	↓	↓	-
C5	↓↓	↓↓	↓↑	↓↑	↓↑	↑	↓	↑	-

**Table:** 

	**Power spectral density**																			
	**PSD: THETA**					**PSD: ALPHA**					**PSD: BETA**					**PSD: GAMMA**				
	**F**	**P**	**O**	**LT**	**RT**	**F**	**P**	**O**	**LT**	**RT**	**F**	**P**	**O**	**LT**	**RT**	**F**	**P**	**O**	**LT**	**RT**
C1	-↑	↓↓	↓↓	↓↓	↓↑	↓↓	↓↓	↓↓	↓↓	↑↑	↑↑	↑↑	↑↑	-↑	↑↑	↑↑	↑↑	↓-	↓↓	↓↓
C2	↑↑	↑↑	↑↑	↑↑	↑↑	↓↑	-↑	-↑	↑↑	↑↑	↑↑	↑↑	↑↑	↑↑	-↑	↓↓	↓↓	↓↓	↓↓	↓↓
C3	↑↑	↑↑	↑↑	↑↑	↑↑	↑↑	↑↑	↑↑	↑↑	↓↓	↑↑	↑↑	↑↑	↑↑	↑↑	↑↑	↑↑	↑↑	↑↑	↑↑
C4	↓↓	↓↓	↓↓	↓↑	↓↓	↓-	–	-↑	↑↑	–	↓↓	↓↓	↓↓	↑↑	-↓	↓↓	↓-	↓↓	↓↑	↓↓
C5	↓↓	↓↓	↓↓	↓↓	↓↓	↓↓	↓↓	↓↓	↓↓	↓↓	↓↓	↓↓	↓↓	↓↓	↓↓	↓↓	↓↓	↓↓	↓↓	↓↓

*QoLS, Quality of Life Scale; DASS, Depression Anxiety Stress Scale; PSYRATS, Psychotic Symptom Rating Scales*.

Table 4Treatment Group: Values pre and post treatment of N1-P3, Connectivity, Psychometric and Power spectral density of the frequent (blue) and rare (orange) stimuli(F, frontal; P, parietal; O, occipital; LT, left temporal; RT, right temporal).

**Table:** 

	**Connectivity**		**Psychometrics**					
	**Participation coefficient (%)**		**QoL**		**DASS**		**PSYRATS**	
	**Pre**	**Post**	**Pre**	**Post**	**Pre**	**Post**	**Pre**	**Post**
T1	1	9	68	77	8	9	21	16
T2	6	13	55	75	92	85	32	25
T3	10	8	50	69	118	53	34	28
T4	9	10	94	91	5	4	30	23
T5	15	26	93	96	39	58	23	28

**Table:** 

	**Freq**				**P**				**O**				**LT**				**RT**			
	**Pre**		**Post**		**Pre**		**Post**		**Pre**		**Post**		**Pre**		**Post**		**Pre**		**Post**	
	**Freq**	**Rare**	**Freq**	**Rare**	**Freq**	**Rare**	**Freq**	**Rare**	**Freq**	**Rare**	**Freq**	**Rare**	**Freq**	**Rare**	**F**	**Rare**	**F**	**Rare**	**F**	**Rare**
	**N1-P3 amplitude (uV)**																			
T1	3.52	6.91	4.12	7.33	5.05	10.08	4.85	7.85	3.91	5.58	4.37	2.77	6.50	10.49	5.15	8.60	5.25	8.32	6.03	7.42
T2	2.05	6.77	1.61	4.27	1.72	4.73	1.56	5.33	2.10	5.25	1.33	4.43	4.64	9.75	1.02	10.06	3.66	9.33	0.99	8.30
T3	5.24	5.17	5.36	10.14	5.59	8.93	6.68	10.02	5.50	7.03	4.67	5.78	5.98	10.81	5.40	10.79	5.40	9.53	5.18	9.81
T4	3.39	6.60	8.11	12.37	3.31	6.72	7.81	12.22	3.50	5.53	7.23	13.33	5.12	5.96	7.49	14.11	4.11	5.24	7.07	12.93
T5	2.68	4.58	2.47	4.10	3.51	5.06	2.72	5.15	3.82	5.08	3.15	4.48	5.20	6.70	3.42	4.42	5.22	6.21	3.39	4.46
	**Power spectral density : Theta band (μ*V*^2^/*Hz)x10***^**−15**^																			
T1	172	338	105	235	152	244	94	255	145	262	95	208	124	228	109	356	196	225	166	259
T2	59	120	21	170	44	95	15	164	45	145	17	194	66	232	26	208	82	111	22	457
T3	88	130	47	292	59	102	19	139	43	107	17	171	58	66	27	527	77	424	70	599
T4	37	85	348	468	36	78	169	264	39	91	355	639	30	53	427	621	66	102	267	481
T5	112	379	83	317	197	378	44	148	279	386	47	134	113	261	95	322	364	504	69	303
	**Power spectral density : Alpha band (μ*V*^2^/*Hz)x10***^**−15**^**)**																			l
T1	116	395	28	92	100	245	26	90	167	366	30	92	262	548	42	175	221	697	53	100
T2	67	348	40	484	65	217	37	393	66	241	62	490	32	295	61	521	203	340	106	1047
T3	109	304	44	493	84	111	22	176	120	178	43	273	87	278	55	332	300	573	97	413
T4	31	52	39	244	14	21	31	137	35	59	56	170	17	44	45	118	38	109	44	255
T5	24	86	11	45	34	136	9	31	45	198	9	27	19	87	14	32	61	235	17	40
	**Power spectral density : Beta band (μ*V*^2^/*Hz)x10***^**−15**^																			
T1	7	28	6	37	6	19	5	37	15	48	6	40	16	69	7	27	15	44	11	129
T2	21	93	13	50	22	93	13	50	28	127	18	65	33	258	14	50	50	177	31	84
T3	29	138	10	99	20	103	4	59	33	218	8	113	54	405	13	108	43	339	21	282
T4	3	13	31	111	2	10	31	110	4	14	33	110	3	14	40	118	4	16	22	121
T5	9	47	3	8	9	45	3	6	11	46	3	7	11	46	3	11	15	68	3	7
	**Power spectral density : Gamma band (μ*V*^2^/*Hz)x10***^**−15**^																			
T1	0.8	6	3	8	0.5	4	3	7	2	11	3	8	2	14	2	5	0.9	7	5	17
T2	10	46	1	3	11	49	1	3	12	54	1	4	14	59	1	2	14	67	2	5
T3	0.6	3	4	47	0.5	2	2	20	0.8	3	3	33	0.5	3	4	37	0.6	2	6	57
T4	1	3	16	51	1	2	15	52	1	4	19	63	2	4	19	68	2	4	14	52
T5	3	12	1	3	3	15	1	3	3	17	1	4	3	13	2	7	4	19	1	4

*QoLS, Quality of Life Scale; DASS, Depression Anxiety Stress Scale; PSYRATS, Psychotic Symptom Rating Scales*.

Table 5Control Group: Values pre and post treatment of N1-P3, Connectivity, Psychometric and Power spectral density of the frequent (blue) and rare (orange) stimuli(F, frontal; P, parietal; O, occipital; LT, left temporal; RT, right temporal).

**Table:** 

	**Connectivity**		**Psychometrics**					

	**Participation coefficient (%)**		**QoL**		**DASS**		**PSYRATS**	
	**Pre**	**Post**	**Pre**	**Post**	**Pre**	**Post**	**Pre**	**Post**
C1	29	26	83	84	34	26	28	28
C2	11	15	68	69	52	56	31	29
C3	8	16	41	79	80	42	32	31
C4	13	20	86	78	89	81	31	30
C5	1	22	96	82	11	18	30	27

**Table:** 

	**Freq**				**P**				**O**				**LT**				**RT**			
	**Pre**		**Post**		**Pre**		**Post**		**Pre**		**Post**		**Pre**		**Post**		**Pre**		**Post**	
	**Freq**	**Rare**	**Freq**	**Rare**	**Freq**	**Rare**	**Freq**	**Rare**	**Freq**	**Rare**	**Freq**	**Rare**	**Freq**	**Rare**	**F**	**Rare**	**F**	**Rare**	**F**	**Rare**
	**N1-P3 amplitude (μV)**																			
C1	2.94	4.38	2.16	8.27	4.22	4.87	2.71	6.80	2.71	3.09	1.31	7.94	4.61	7.05	0.91	9.58	3.77	6.11	1.49	9.53
C2	8.79	10.26	7.93	10.7	9.54	15.23	8.03	10.98	7.04	7.89	7.67	10.49	5.75	6.22	7.90	14.49	5.84	5.33	7.76	10.88
C3	1.02	3.01	5.16	7.43	2.08	4.59	6.84	8.67	2.01	5.51	5.65	8.42	5.31	11.63	6.89	9.31	5.09	10.61	7.01	9.61
C4	6.49	4.38	1.65	2.15	6.09	5.50	1.63	2.18	5.86	6.83	1.16	1.69	5.97	10.90	1.23	2.01	5.62	10.07	1.22	1.58
C5	5.10	4.71	4.16	2.57	9.16	14.05	4.40	5.72	5.31	5.08	2.62	8.73	8.31	11.93	4.01	13.87	9.00	10.37	4.03	12.49
	**Power spectral density : Theta band (μ*V*^2^/*Hz)x10***^**−15**^																			
C1	46	154	68	381	42	146	62	192	45	141	51	195	78	129	59	167	46	118	76	382
C2	647	1651	544	792	245	606	299	289	153	506	188	264	571	1484	156	754	212	1933	311	459
C3	34	42	61	85	29	48	53	79	44	64	73	105	14	86	40	150	80	137	119	102
C4	653	1580	38	175	506	1430	37	190	688	2157	78	216	577	3253	231	328	793	2011	38	225
C5	660	763	429	1919	748	847	281	1543	945	1078	317	1453	1759	1273	1425	3293	1251	1098	200	2306
	**Power spectral density : Alpha band (μ*V*^2^/*Hz)x10***^**−15**^																			
C1	34	120	51	215	44	85	35	129	40	116	43	181	46	259	69	174	26	97	45	112
C2	165	849	196	207	97	304	142	156	152	227	174	191	424	563	199	187	162	756	120	325
C3	39	105	79	141	27	94	73	106	34	135	90	153	17	50	130	185	48	345	62	219
C4	139	229	10	73	66	188	10	79	116	238	24	170	306	580	87	391	131	192	10	85
C5	54	177	78	220	57	153	61	218	63	204	77	247	59	340	66	353	55	282	100	347
	**Power spectral density : Beta band (μ*V*^2^/*Hz)x10***^**−15**^																			
C1	6	16	9	61	5	17	8	47	5	23	11	58	6	40	18	102	6	24	13	70
C2	18	32	34	29	8	16	21	17	6	19	13	20	17	27	22	43	15	57	28	28
C3	5	11	11	22	5	13	10	22	4	12	12	33	5	10	17	47	5	16	11	23
C4	25	102	4	11	16	60	4	11	23	83	6	23	38	178	14	40	21	76	6	12
C5	8	18	15	58	7	14	12	54	7	14	15	66	7	15	36	190	8	21	10	56
	**Power spectral density : Gamma band (μ*V*^2^/*Hz)x10***^**−15**^																			
C1	2	7	3	21	2	5	2	20	3	8	3	21	5	23	6	48	4	9	3	18
C2	8	20	0.5	0.4	6	16	0.3	0.3	6	18	0.2	0.3	9	24	0.4	0.5	8	27	0.3	0.3
C3	3	7	6	24	3	8	6	24	3	9	8	30	6	19	13	42	4	11	5	27
C4	3	19	0.2	2	3	14	0.2	2	4	28	0.2	2	7	41	0.5	8	7	27	0.3	2
C5	2	4	1	7	2	4	1	5	1	4	1	7	2	4	4	13	3	7	1	5

*QoLS, Quality of Life Scale; DASS, Depression Anxiety Stress Scale; PSYRATS, Psychotic Symptom Rating Scales*.

### 3.2. Study Case 1 : Improvement in Psychometric Score

**Figure 3:**
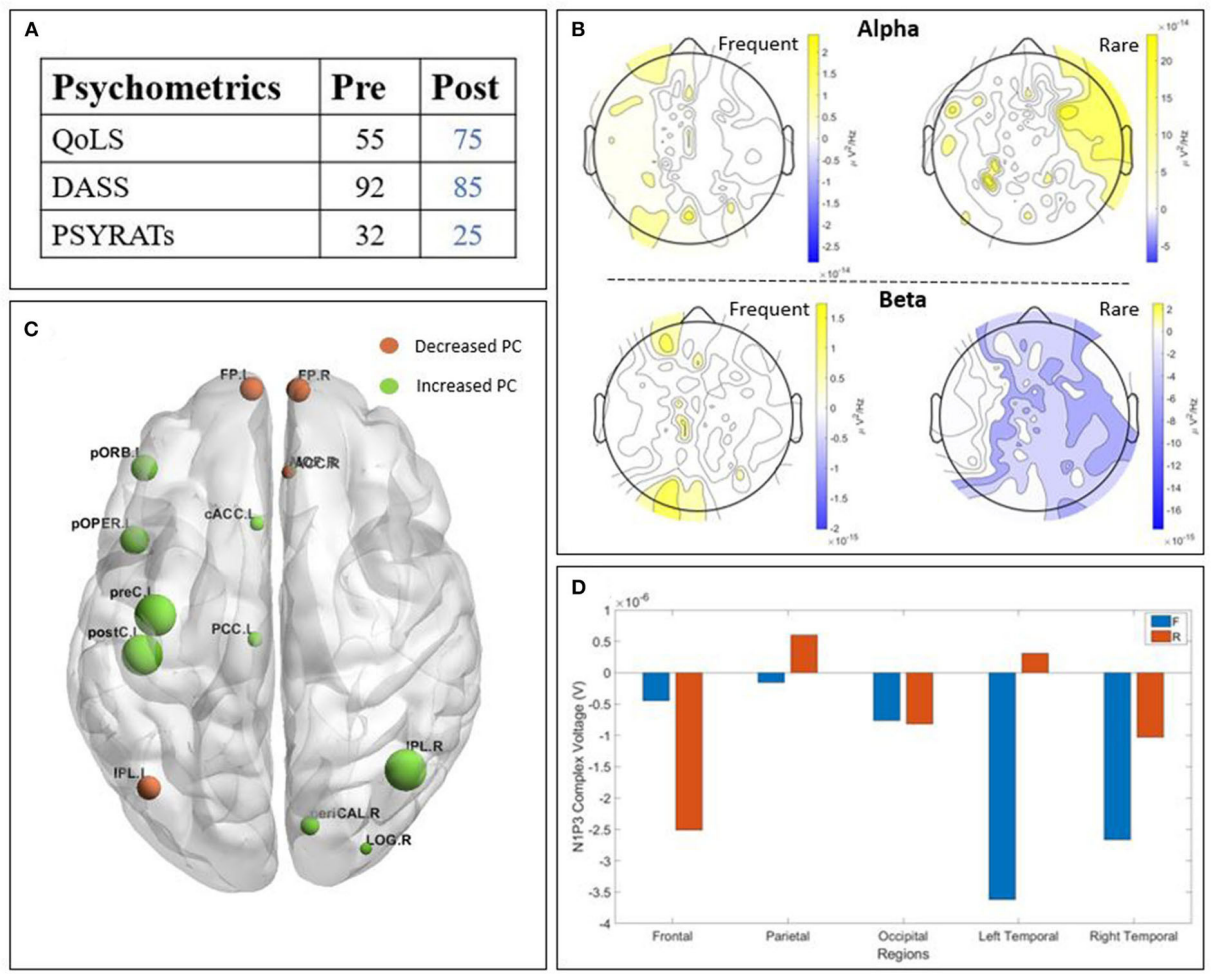
Results of patient T2 : **(A)** psychometric; **(B)** Scalp-level frequency analysis; **(C)** Source-space connectivity; **(D)** Scalp-level time analysis. The yellow areas in frequency analysis are related to a higher Power Spectral Density (PSD) post-treatment, whereas the blue ones are related to a higher PSD pre-treatment. The size of the node in the connectivity is related to the amount of increase (green) or decrease (orange) participation coefficient (PC) values. The positive bars in time analysis are related to a higher N1-P3 amplitude post-treatment. (QoLS, Quality of Life Scale; DASS, Depression Anxiety Stress Scale; PSYRATS, Psychotic Symptom Rating Scales).

**Figure 4:**
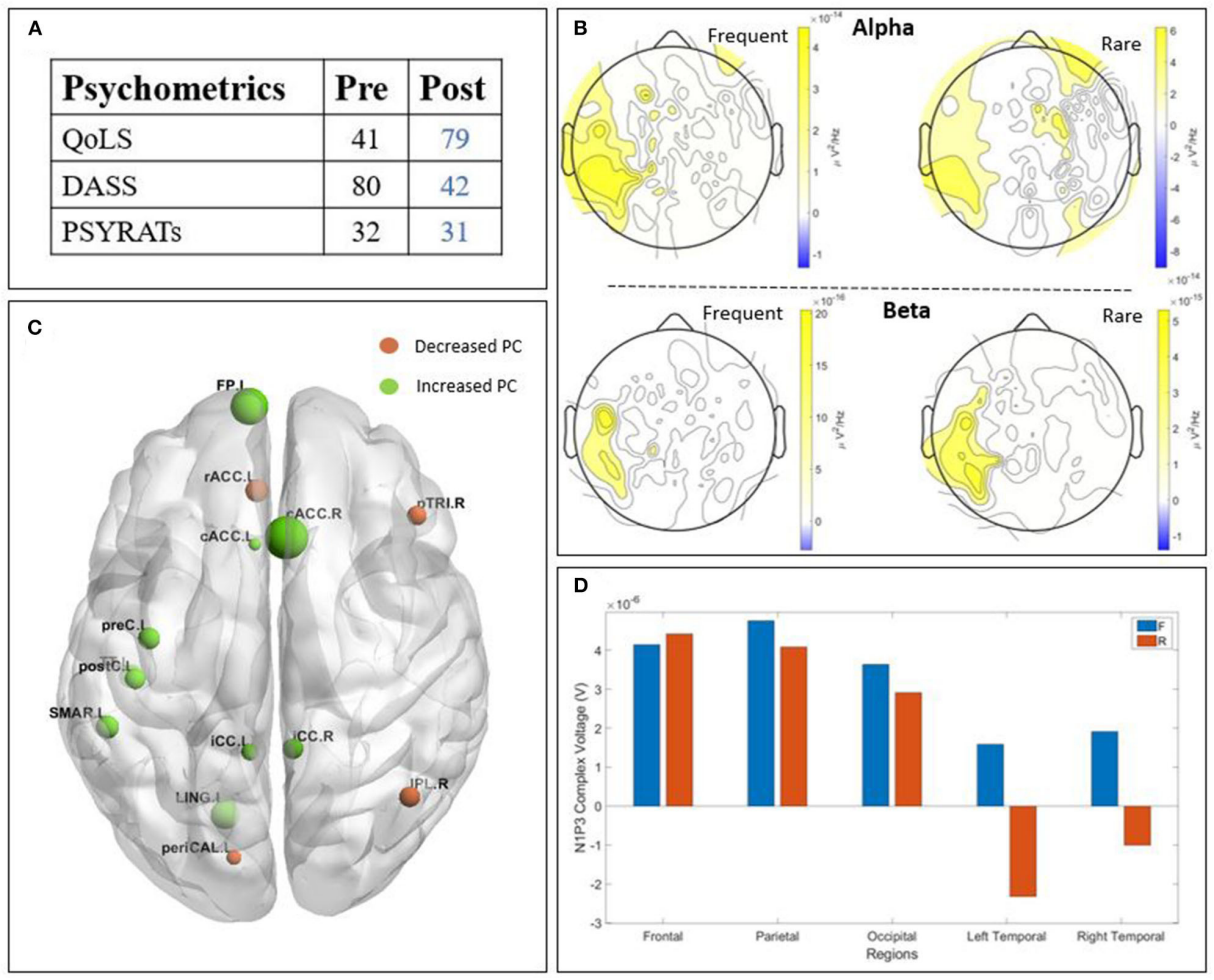
Results of patient C3 : **(A)** psychometric; **(B)** Scalp-level frequency analysis; **(C)** Source-space connectivity; **(D)** Scalp-level time analysis. The yellow areas in frequency analysis are related to a higher Power Spectral Density (PSD) post-treatment, whereas the blue ones are related to a higher PSD pre-treatment. The size of the node in the connectivity is related to the amount of increase (green) or decrease (orange) participation coefficient (PC) values. The positive bars in time analysis are related to a higher N1-P3 amplitude post-treatment. (QoLS, Quality of Life Scale; DASS, Depression Anxiety Stress Scale; PSYRATS, Psychotic Symptom Rating Scales).

The two patients detailed in this section presented an improvement in their psychometric score post-treatment. We chose to describe them in this section due to their higher values post treatment in the neurophysiological data, (Figures 3A, 4A) in order to find a potential correlation between those two outcomes.

#### 3.2.1. Patient T2

Patient T2 (Figure 3) is a man with paranoid schizophrenia, in the TG, who took part in the study while taking : clozapine, olanzapine, perphenazine, alprazolam, levomepromazine, oxazepam, and melatonin. The psychometric tests (Figure 3A) show an improvement of the quality of life post-treatment, a decreased DASS after TMS and decreased PSYRATS post-treatment. The temporal analysis (Figure 3D) showed a lower N1-P3 amplitude post-treatment, except for the parietal and left temporal parts. The PSD (Figure 3B) showed higher alpha power post-TMS. However, the beta power is lower post-TMS. The connectivity (Figure 3C) revealed a clear higher participation coefficient (represented by the larger green nodes), especially in the left central, left orbito-frontal and the right occipital brain regions. The frontal area showed a relatively lower participation coefficient.

#### 3.2.2. Patient C3

Patient C3 (Figure 4) is a woman with paranoid schizophrenia, in the CG, who tool part in the study while taking : aripriprazole, olanzapine, chloroprothixene, and pregabalin. The psychometric outcome (Figure 4A) revealed an improvement after the treatment. The quality of life increased, the DASS decreased, while the PSYRATS did not change. The time domain analysis (Figure 4D) showed a higher amplitude of the N1-P3 complex after the treatment, except on the temporal regions for the rare stimulus. The PSD (Figure 4B) showed higher alpha power post-TMS, except from the right temporal region for both frequent and rare stimuli. The beta band also showed a higher PSD post-TMS. Finally, the connectivity study (Figure 4C) displayed a globally improved participation coefficient, principally in the frontal, occipital, and central areas of the brain.

### 3.3. Study Case 2 : Stagnation in Psychometric Score

**Figure 5:**
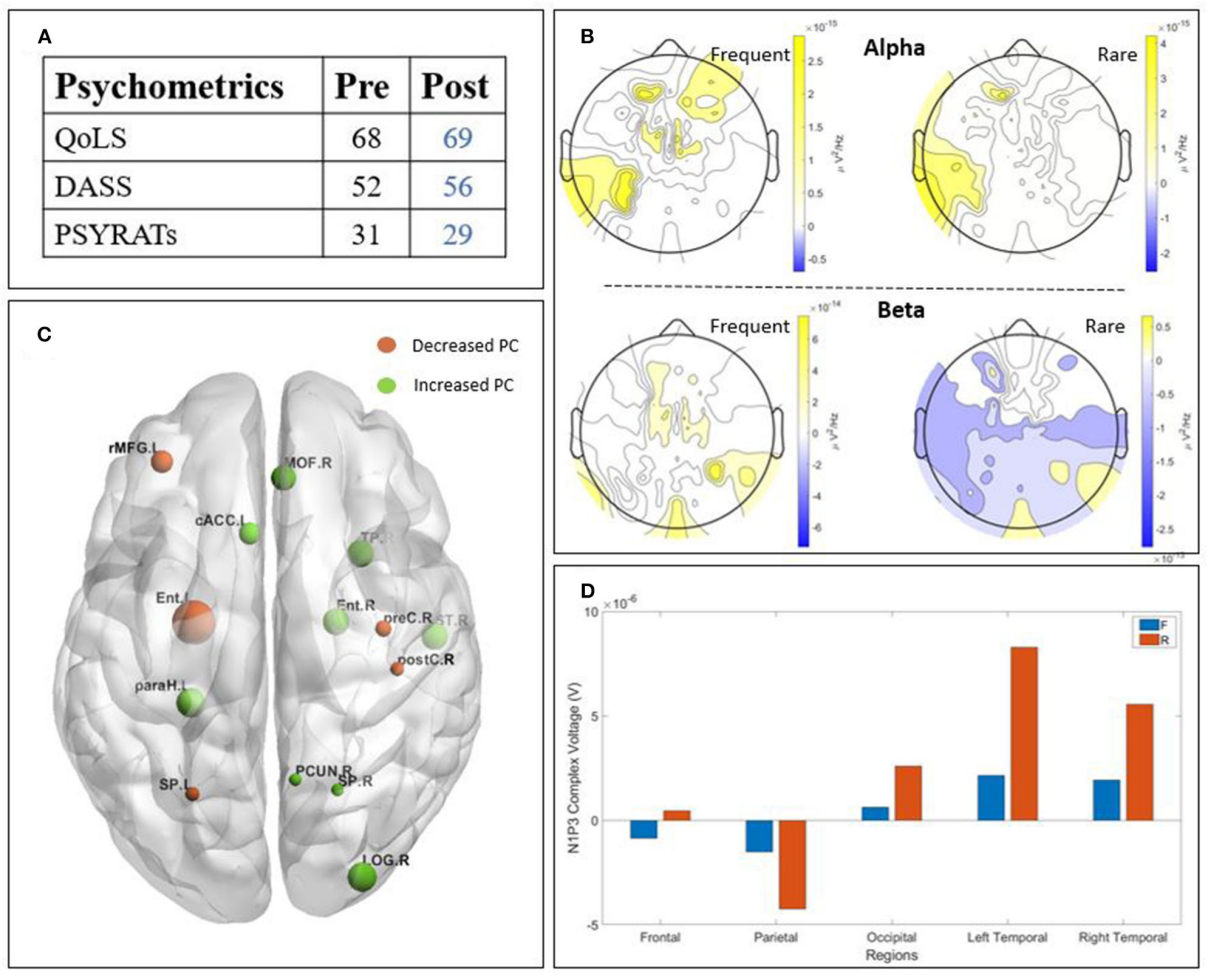
Results of patient C2 : **(A)** psychometric; **(B)** Scalp-level frequency analysis; **(C)** Source-space connectivity; **(D)** Scalp-level time analysis. The yellow areas in frequency analysis are related to a higher Power Spectral Density (PSD) post-treatment, whereas the blue ones are related to a higher PSD pre-treatment. The size of the node in the connectivity is related to the amount of increase (green) or decrease (orange) participation coefficient (PC) values. The positive bars in time analysis are related to a higher N1-P3 amplitude post-treatment. (QoLS, Quality of Life Scale; DASS, Depression Anxiety Stress Scale; PSYRATS, Psychotic Symptom Rating Scales).

The patient detailed in this section presented a stagnation in her psychometric score post treatment (Figure 5A). We chose to describe her in this section in order to find a potential correlation with the neurophysiological data.

#### 3.3.1. Patient C2

Patient C2 (Figure 5) is a woman with paranoid schizophrenia, in the CG, who took part in the study while taking : clozapine, flupenthixol, zopiclone, mirtazapine, escitalopram, metoprolol, and chlorpomazine. The psychometric data (Figure 5A) showed that the treatment did not have a lot of impact on this scale. The quality of life, the DASS and the PSYRATS remained more or less the same. The time domain (Figure 5D) showed a global increase of N1-P3 amplitude post-TMS, except for the parietal region for both stimuli and the frontal region for the frequent stimulus. The PSD analysis (Figure 5B) showed higher alpha and beta power post-TMS (with the exception of frontal frequent stimulus responses in the alpha band). The connectivity analysis (Figure 5C) revealed a balanced participation evolution. Globally the left hemisphere (mainly the entorhinal and frontal) showed a decreased participation coefficient, and the right areas (mainly the frontal and occipital) showed an increased participation coefficients.

### 3.4. Study Case 3 : Decrease in Psychometric Score

**Figure 6:**
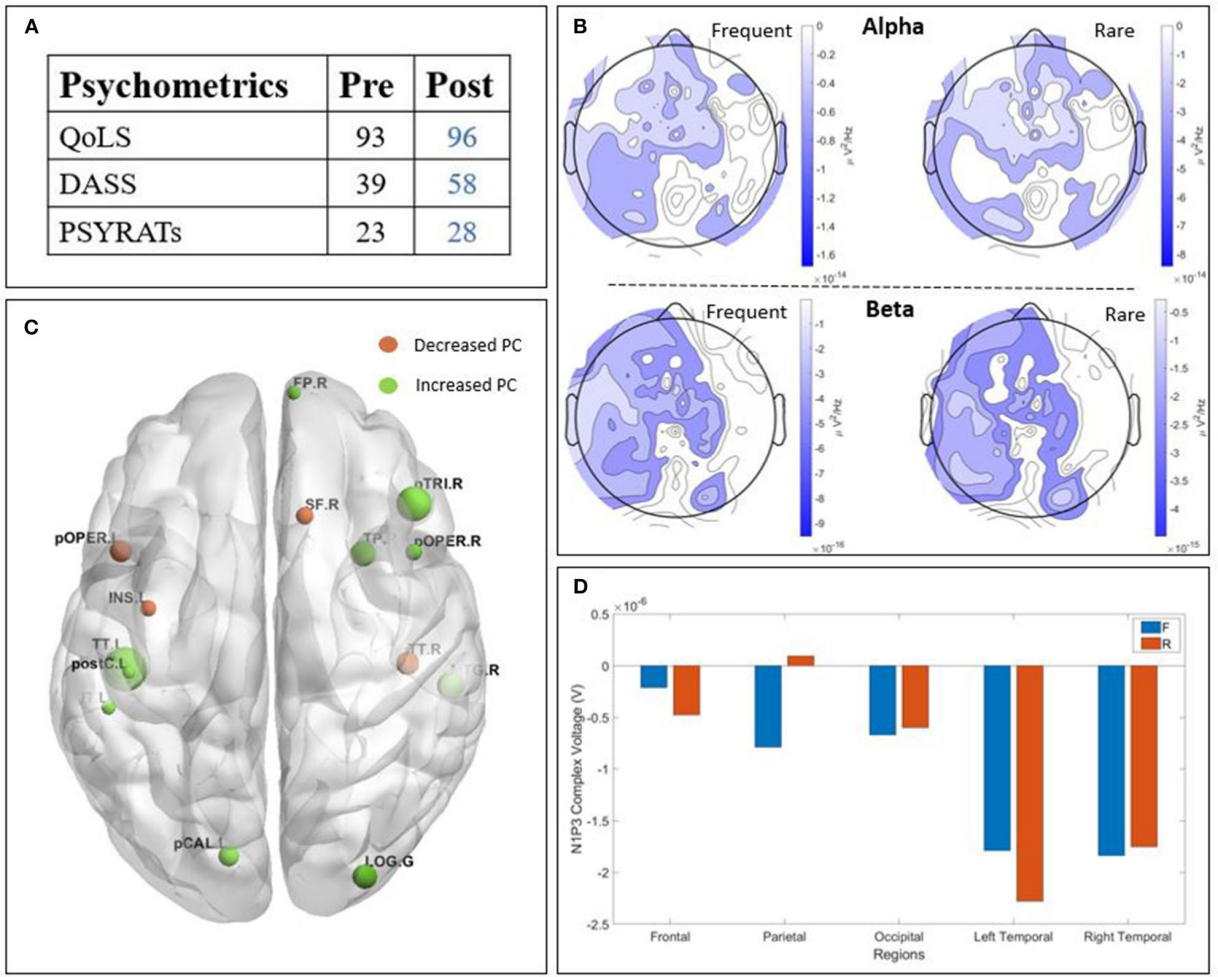
Results of patient T5 : **(A)** psychometric; **(B)** Scalp-level frequency analysis; **(C)** Source-space connectivity; **(D)** Scalp-level time analysis. The yellow areas in frequency analysis are related to a higher Power Spectral Density (PSD) post-treatment, whereas the blue ones are related to a higher PSD pre-treatment. The size of the node in the connectivity is related to the amount of increase (green) or decrease (orange) participation coefficient (PC) values. The positive bars in time analysis are related to a higher N1-P3 amplitude post-treatment. (QoLS, Quality of Life Scale; DASS, Depression Anxiety Stress Scale; PSYRATS, Psychotic Symptom Rating Scales).

The patient detailed in this section presented a decrease in their psychometric score post treatment. We chose to describe him due to his lower values in the neurophysiological data post treatment (Figure 6A), in order to find a potential correlation between those two outcomes.

#### 3.4.1. Patient T5

Patient T5 (Figure 6) is a man with paranoid schizophrenia, in the TG, who took part in the study while taking : clozapine, flupenthixol, zopiclone, mirtazapine, escitalopram, metoprolol, and chlorpomazine. The psychometric data (Figure 6A) showed very little effect of treatment on this scale. The quality of life remained the same, the DASS increased and the PSYRATS slightly increased. The time domain (Figure 6D) showed a global decrease of N1-P3 amplitude post-TMS, except for the parietal region for the rare stimulus. The PSD analysis (Figure 6B) showed a lower alpha power post-TMS, except for the right temporal region. The beta power decreased as well, except for the right temporal region for the frequent stimulus. The connectivity analysis (Figure 6C) showed a globally higher participation coefficient in the right frontal, left central, and occipital brain regions.

## 4. Discussion

The present work aimed to develop hypothesis to assess the effects of TMS in schizophrenia with AVH, analysing EEG data and psychometric outcome. This was based on three different approaches : temporal size (with the calculation of N1P3 complex amplitude), spectral [with the evaluation of the PSD in several frequency bands (theta, alpha, beta, and gamma)] and connectivity, (with the calculation of the participation coefficient in beta and gamma band).

The general results from our study revealed a high variability between individuals, in both groups. This can be explained in several ways : Firstly, subjects were taking a range of medications all of which can interfere with the background neural activity and the generation of ERPs (Javitt et al., 2008). Secondly, the long and tiring recording procedure (around 1 h), and the different states of the patients during the protocol could also have led to varying data quality. Indeed, Polich (1997) highlights the fact that background EEG variation contributes significantly to a high P300 individual variability.

However, there were some indications of improvement in the psychometric results, in four out of five subjects in TG and three out of five in CG. Half of our subjects (six out of 10, four CG, two TG) showed an increase of the N1-P3 amplitude after the treatment, especially for the rare stimulus. Bramon et al. (2004) and Jeon and Polich (2003) established that patients with schizophrenia and AVH presented an inhibition to the P300 experiment. Thus, the clearer presence of N100 and P300 waves post-TMS, which is a known response to the auditory oddball task (Patel and Azzam, 2005), suggested a response to TMS. Likewise, the spectral analysis displayed an increase of the PSD in alpha and beta bands for six subjects (4 in CG, 2 in TG). Ray and Cole (1985) demonstrated that those bands were directly linked to attention, focus, emotional and cognitive processes. A higher power in these bands could be indicative of a change in those mechanisms. Finally, our brain connectivity results showed a global increased participation coefficient in the beta band after treatment, for six subjects (3 in TG, 4 in CG). These results lead us to the conclusion that TMS seemed to have a positive impact on the patients, in both groups. However, it is not possible to assume that the location where the treatment was applied had a different impact on the brain function. The results in connectivity analysis show indeed an improved participation coefficient thus a better network integration independently from the group. Therefore, we discussed the results without regard to the patients' group. We chose to underline in the results section some patients that showed an agreement between the psychometric scores and our neurophysiological data.

It is interesting to note that for patient C3, where the psychometric results were better post-TMS, all three neurophysiological components used for this study revealed a higher value post-TMS. For this patient, there seems to be a clear link between clinical and neurophysiological outcomes. For T2, the trend is there, but is less obvious, with half of the neurophysiological results being in concordance with the improved psychometric score. Conversely, patient T5 had a deterioration in their psychometric post-TMS, and the same tendency is visible in their neurophysiological data.

Although there was significant dissociation between clinical and neurophysiological outcome, the participation coefficient from the connectivity analysis was the parameter that seemed to interact most closely with the psychometric results, followed by alpha power. Concerning the connectivity analysis, our results showed an increased network integration in some brain regions and a slight decrease in other regions, different for every patient. This reconfiguration of the brain network has been widely reported in the literature when stimulating the brain using electrical and magnetic means, and is also present in several brain disorders (Fornito et al., 2015). The increased network integration may be related to better information processing in the human brain and more efficient networks. This increase in network integration was associated with a decrease in this same integration in other brain regions, reflecting the inter-subject variability. Although the small sample size did not allow us to test statistical significance, we showed a clear “trend” of reshaping of the functional brain network between the different conditions.The frequency analysis revealed the most interesting changes in power spectrum, mainly in the alpha and beta bands. There was higher alpha power post-TMS, which is less prominent but still visible in the beta power. The gamma and theta power did not show any clear trends. The fact that alpha and beta bands are directly linked to attention, focus and emotional tasks (Ray and Cole, 1985) is interesting. A higher power post-TMS linked with a better score in the psychometric scale could indicate that the improvement of these cognitive mechanisms was directly linked to the progress of the patient's clinical condition. Due to our small sample size, this has to be put in perspective, and nothing definitive can be assessed. However, there was a “trend” of an increase in alpha and beta powers post-TMS that is linked with an improvement in the clinical outcome. Finally, the time domain was the area of analysis presenting the most variability. Half of the patients presented a higher N1-P3, especially in the rare stimuli, but no clear trend was established between this outcome and the clinical outcome. However, due to its conclusive results in studies related to schizophrenia with AVH (Ogura et al., 1991; Ford et al., 2001; Earls et al., 2016), our small number of subjects, as well as its subject inter-variability (Polich, 1997), it is a paradigm that should be taken in account in further studies.

Considering the fact that psychometric tests are a semi-self assessment evaluation of patients condition, this work is a first step toward a development of a hypothesis to correlate and validate psychometrics with quantitative neurophysiological data. We suggest further investigation of any link between psychometrics and neurophysiological data under the umbrella of TMS, focusing mainly on the participation coefficient in the beta band and the power spectral density in alpha band. The beta power as well as the N1-P3 amplitude should also be considered of interest.

### 4.1. Limitations

The study has many limitations. Firstly, due to the very small sample size, it was not possible to assess our results definitively. Rather, we aimed to discover trends in order to generate hypotheses for further study. Secondly, the patients were also undergoing their usual treatment, including antipsychotic and sedative medications. This did not change between pre- and post-rTMS conditions but might have influenced the background neural activity and the generation of the ERPs (Javitt et al., 2008). Thirdly, the experimental procedure was long (1 h) and tiring and some patients had difficulty cooperating and maintaining task engagement, which may have affected data quality. Muscle and movement artifacts added noise to the EEG signal, requiring a thorough pre-processing and the exclusion of many trials. We encourage similar experiments with patients in the supine position to reduce the noise and improve the data quality, making them easier to process and analyze. Finally, in this study we only analyzed the oddball auditory paradigm. We recommend pursuing this work using other procedures as well, in order to have a more complete overview of the results.

### 4.2. Future Directives

Future studies of rTMS and neurophysiological markers in schizophrenia should recruit larger number of participants than the present study. The possible association of AVH and other symptoms of schizophrenia with variations in P300, PSD and other EEG markers should be studied further. This may help to establish whether the PSD in the alpha and beta bands, the N1-P3 complex and the participation coefficient in beta bands are reliable biomarkers of the neural response to TMS in patients with schizophrenia and AVH.

## 5. Conclusion

After conducting TMS, most patients showed an evolution in psychometric data as well as on the neurophysiological quantitative data, independent of the stimulation site. We examined the interplay between the psychometric and the neurophysiological data. When the psychometric improved post-TMS, we could observe an increased network integration mainly, through the participation coefficient in the beta bands, a higher alpha and beta band power, and sometimes a higher N1-P3 amplitude. Due to the small sample size, it is not possible to assess definitively the impact of TMS on the brain function in schizophrenia, nor the correlation between psychometric and neurophysiological data. However, our results suggest that brain connectivity, through the participation coefficient, alpha and beta power bands, were highly related with the psychometric score, and that N1-P3, despite his variability, should be investigated. This hypothesis will have to be verified in further studies, with a larger sample size, and an improved recording procedure, leading to a better data quality. This is a first step toward the definition of quantitative neurophysiological parameters to assess TMS treatment.

## Data Availability Statement

The raw data supporting the conclusions of this article will be made available by the authors, without undue reservation.

## Ethics Statement

The studies involving human participants were reviewed and approved by Health Research Ethics Committee at the University Hospital of Iceland (approval no. 21.2018). The patients/participants provided their written informed consent to participate in this study.

## Author Contributions

RA and RS wrote the manuscript and performed temporal and spectral group analyses with support from PG. OB, SS, AJ, and EÍ carried out the rTMS treatment. RA, VJ, AJ, EÍ, and OB performed the oddball auditory experiments and HD-EEG acquisitions. OB and EW designed the TMS protocol and made additions to the manuscript. MHas and SY performed the brain connectivity analysis and made additions to the manuscript. MHar and BM designed the psychometrics study. VJ conducted the psychometrics interview. OB and VJ performed psychometrics analysis. DJ reviewed and made additions to the manuscript. OB and PG conceived the original idea and PG coordinated the work. All authors contributed to the article and approved the submitted version.

## Conflict of Interest

The authors declare that the research was conducted in the absence of any commercial or financial relationships that could be construed as a potential conflict of interest.

## Abbreviations

AHS: Auditory Hallucinations Subscale
AVH: Auditory Verbal Hallucinations
CG: Control Group
DASS: Depression Anxiety Stress Scale
ERP: Event-related Potential
HD EEG: High Density Electroencephalogram
PC: Participation coefficient
PSD: Power Spectral Density
PSYRATS: Psychotic Symptom Rating Scales
QoLS: Quality of Life Scale
rTMS: Repetitive Transcranial Magnetic Stimulation
TG: Treatment Group
TMS: Transcranial Magnetic Stimulation.
