# Supplementary material for: P300 Analysis Using High-Density EEG to Decipher Neural Response to rTMS in Patients With Schizophrenia and Auditory Verbal Hallucinations
Source: Front Neurosci. 2020 Nov 20;14:575538. doi: 10.3389/fnins.2020.575538 (PMC7720634; doi:10.3389/fnins.2020.575538)
Supplement: Supplementary file 1 [file Data_Sheet_1.PDF]

Supplementary Material

1 SUPPLEMENTARY TABLES AND FIGURES

1.1 Figures

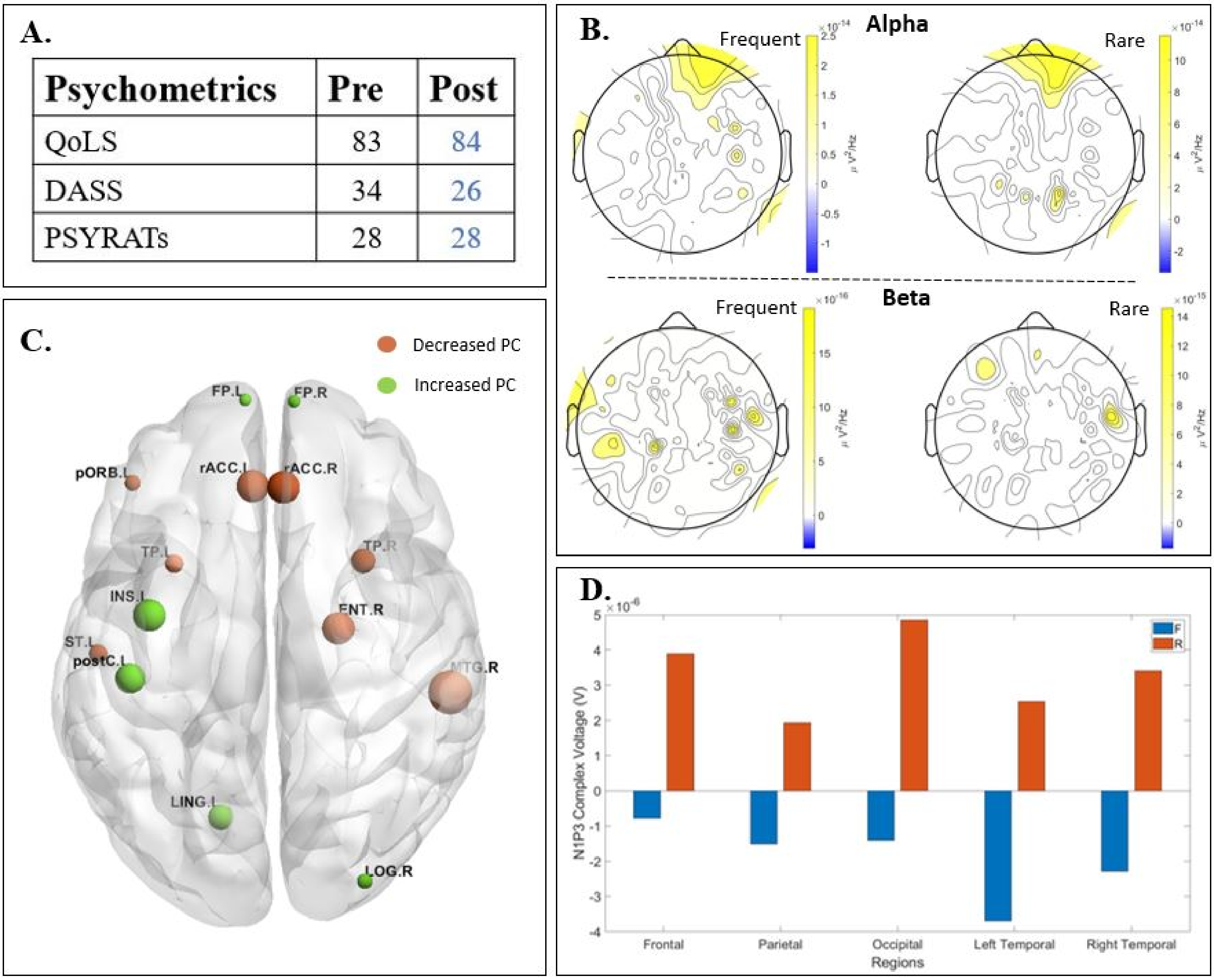

Figure S1. Results of patient C1 : A. psychometric; B. Scalp-level frequency analysis; C. Source-space connectivity; D. Scalp-level time analysis. The yellow areas in frequency analysis are related to a higher Power Spectral Density (PSD) post-treatment, whereas the blue ones are related to a higher PSD pre-treatment. The size of the node in the connectivity is related to the amount of increase (green) or decrease (orange) participation coefficient (PC) values. The positive bars in time analysis are related to a higher N1-P3 amplitude post-treatment. (QoLS: Quality of Life Scale,DASS: Depression Anxiety Stress Scale ,PSYRATS: Psychotic Symptom Rating Scales)

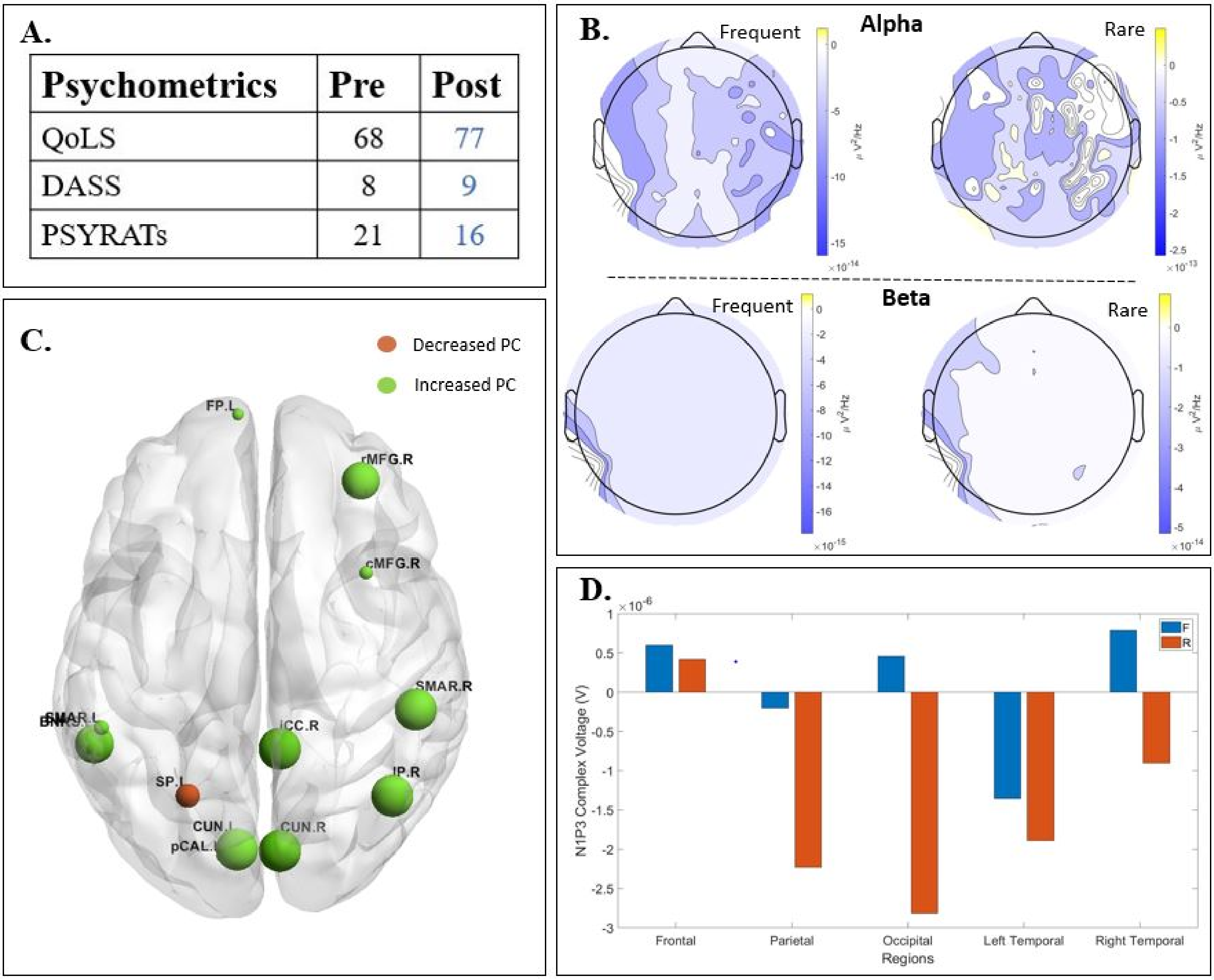

Figure S2. Results of patient T1 : A. psychometric; B. Scalp-level frequency analysis; C. Source-space connectivity; D. Scalp-level time analysis. The yellow areas in frequency analysis are related to a higher Power Spectral Density (PSD) post-treatment, whereas the blue ones are related to a higher PSD pre-treatment. The size of the node in the connectivity is related to the amount of increase (green) or decrease (orange) participation coefficient (PC) values. The positive bars in time analysis are related to a higher N1-P3 amplitude post-treatment. (QoLS: Quality of Life Scale,DASS: Depression Anxiety Stress Scale ,PSYRATS: Psychotic Symptom Rating Scales)

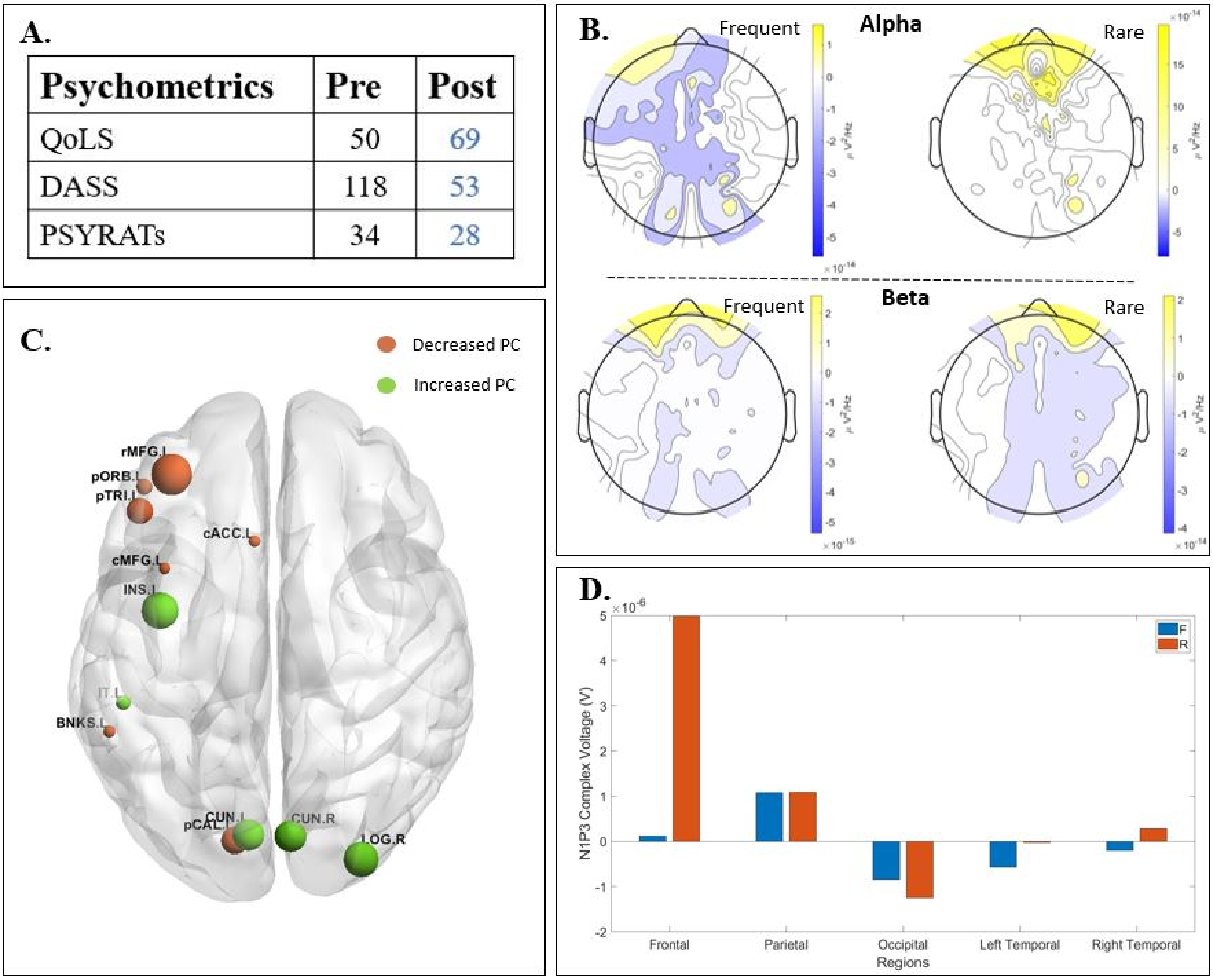

Psychometrics	Pre	Post
QoLS	50	69
DASS	118	53
PSYRATs	34	28

Figure S3. Results of patient T3 : A. psychometric; B. Scalp-level frequency analysis; C. Source-space connectivity; D. Scalp-level time analysis. The yellow areas in frequency analysis are related to a higher Power Spectral Density (PSD) post-treatment, whereas the blue ones are related to a higher PSD pre-treatment. The size of the node in the connectivity is related to the amount of increase (green) or decrease (orange) participation coefficient (PC) values. The positive bars in time analysis are related to a higher N1-P3 amplitude post-treatment. (QoLS: Quality of Life Scale,DASS: Depression Anxiety Stress Scale ,PSYRATS: Psychotic Symptom Rating Scales)

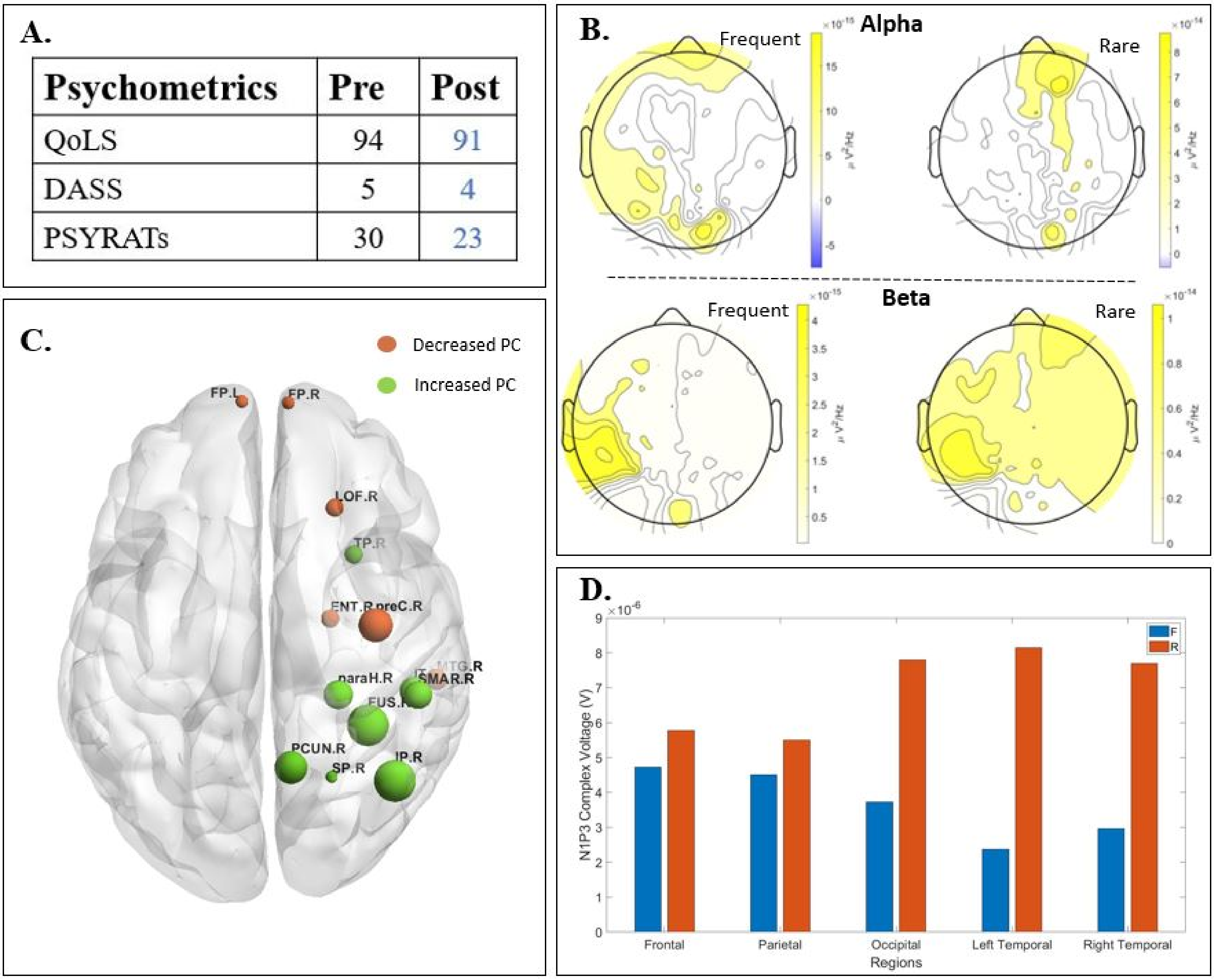

Psychometrics	Pre	Post
QoLS	94	91
DASS	5	4
PSYRATs	30	23

Figure S4. Results of patient T4 : A. psychometric; B. Scalp-level frequency analysis; C. Source-space connectivity; D. Scalp-level time analysis. The yellow areas in frequency analysis are related to a higher Power Spectral Density (PSD) post-treatment, whereas the blue ones are related to a higher PSD pre-treatment. The size of the node in the connectivity is related to the amount of increase (green) or decrease (orange) participation coefficient (PC) values. The positive bars in time analysis are related to a higher N1-P3 amplitude post-treatment. (QoLS: Quality of Life Scale,DASS: Depression Anxiety Stress Scale ,PSYRATS: Psychotic Symptom Rating Scales)

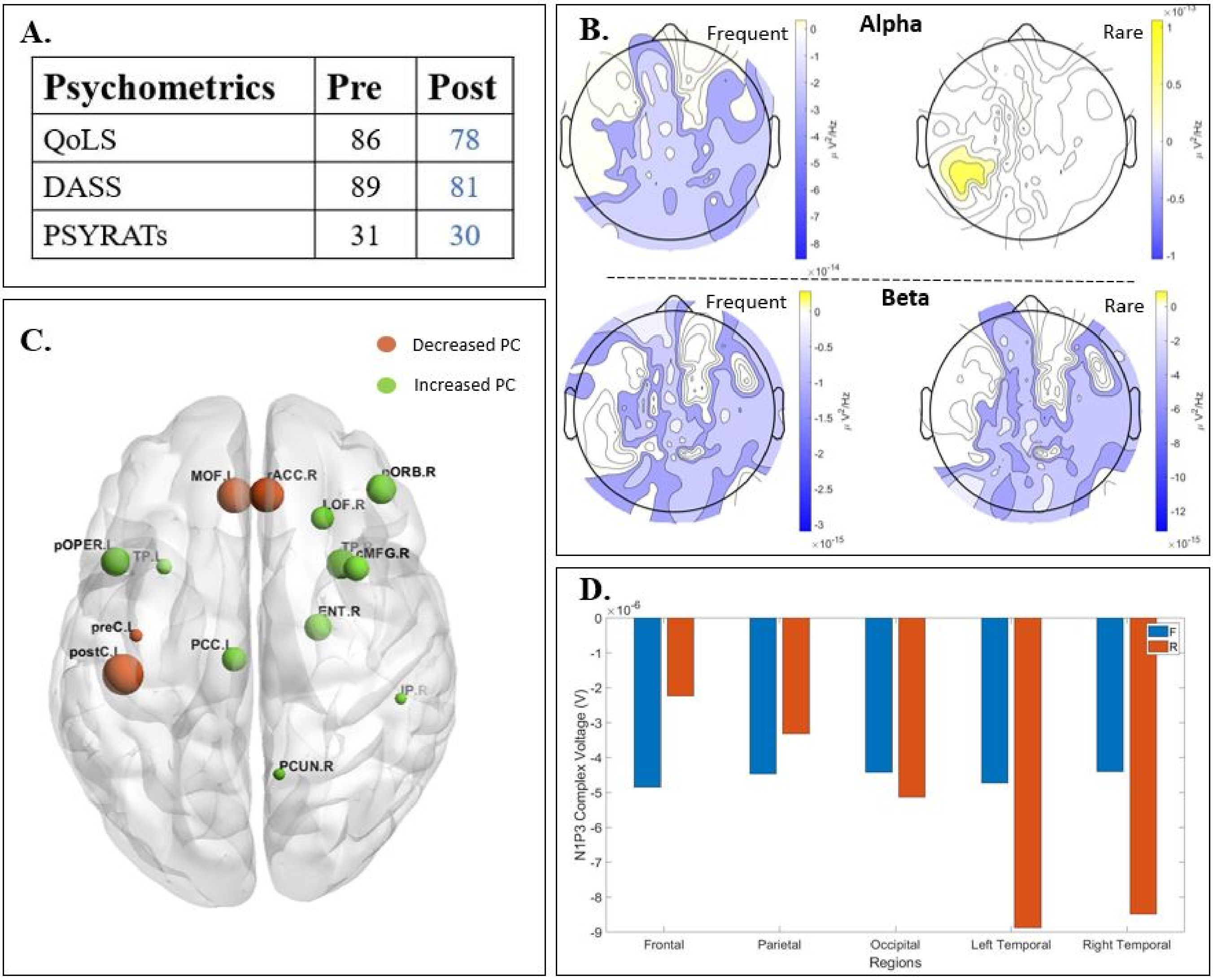

Figure S5. Results of patient C4 : A. psychometric; B. Scalp-level frequency analysis; C. Source-space connectivity; D. Scalp-level time analysis. The yellow areas in frequency analysis are related to a higher Power Spectral Density (PSD) post-treatment, whereas the blue ones are related to a higher PSD pre-treatment. The size of the node in the connectivity is related to the amount of increase (green) or decrease (orange) participation coefficient (PC) values. The positive bars in time analysis are related to a higher N1-P3 amplitude post-treatment. (QoLS: Quality of Life Scale,DASS: Depression Anxiety Stress Scale ,PSYRATS: Psychotic Symptom Rating Scales)

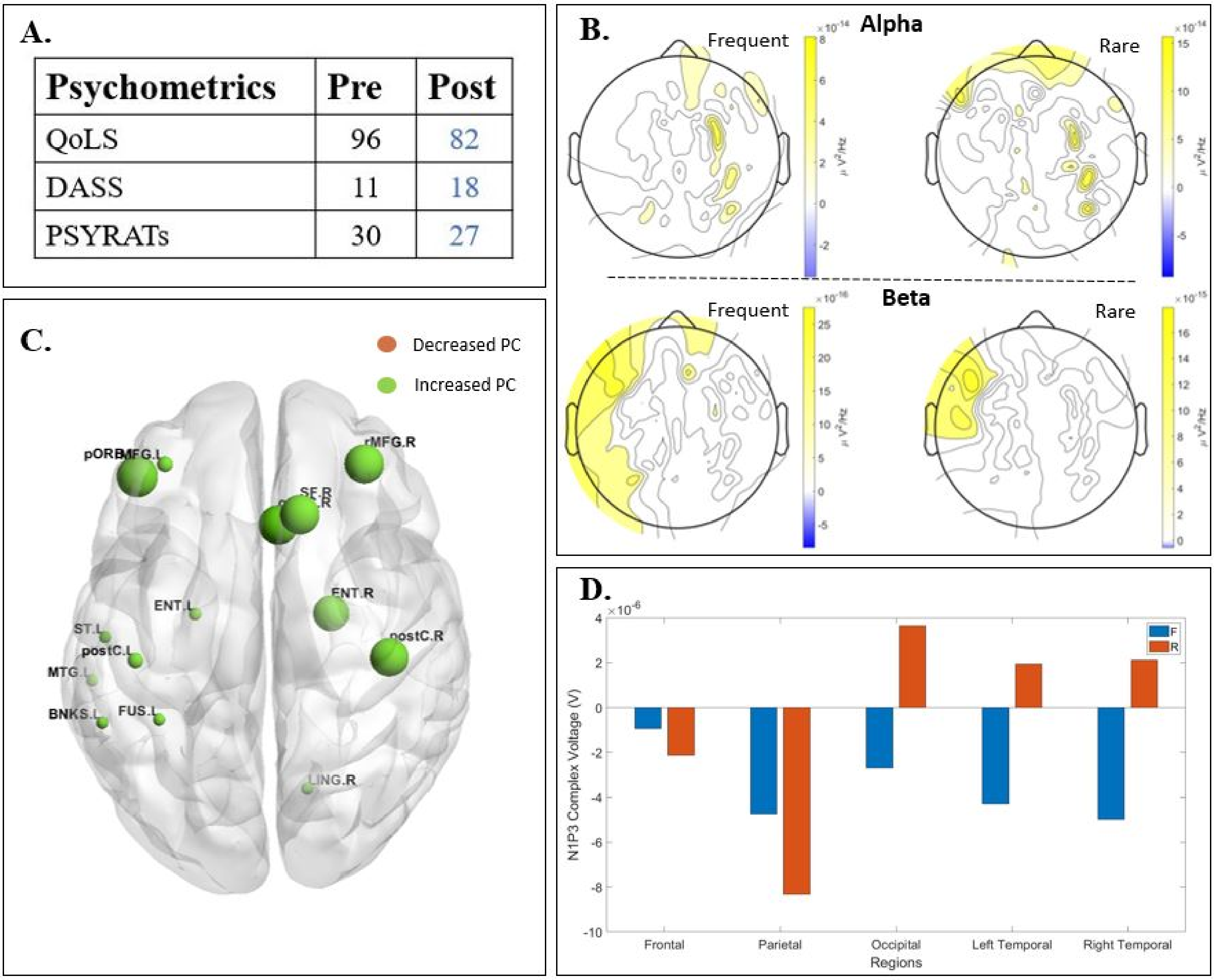

Psychometrics	Pre	Post
QoLS	96	82
DASS	11	18
PSYRATs	30	27

Figure S6. Results of patient C5 : A. psychometric; B. Scalp-level frequency analysis; C. Source-space connectivity; D. Scalp-level time analysis. The yellow areas in frequency analysis are related to a higher Power Spectral Density (PSD) post-treatment, whereas the blue ones are related to a higher PSD pre-treatment. The size of the node in the connectivity is related to the amount of increase (green) or decrease (orange) participation coefficient (PC) values. The positive bars in time analysis are related to a higher N1-P3 amplitude post-treatment. (QoLS: Quality of Life Scale,DASS: Depression Anxiety Stress Scale ,PSYRATS: Psychotic Symptom Rating Scales)
